# Supplementary material for: Novel Pituitary Actions of TAC4 Gene Products in Teleost
Source: Int J Mol Sci. 2021 Nov 29;22(23):12893. doi: 10.3390/ijms222312893 (PMC8657723; doi:10.3390/ijms222312893)
Supplement: Supplementary file 1 [file ijms-22-12893-s001.zip › ijms-1439673-SI.pdf]

SUPPLEMENTARY INFORMATION

Novel pituitary actions of TAC4 gene products in teleost

Xuetao Shi[1#], Cheng Ye[1#], Xiangfeng Qin[1], Lingling Zhou[1], Chuanhui Xia[1], Tianyi Cai[1], Yunyi Xie[1], Zhan Yin[2*], Guangfu Hu[1,2*]

[1]College of Fisheries, Hubei Province Engineering Laboratory for Pond Aquaculture, Huazhong Agricultural University, Wuhan, 430070, China

[2]State Key Laboratory of Freshwater Ecology and Biotechnology, Institute of Hydrobiology, Chinese Academy of Sciences, Wuhan 430072, China

Supplementary Table S1. Primer sequences used for quantitative real-time PCR of TAC4 and NKRs in grass carp

Gene	Forward primer	Reverse primer	Product size (bp)	Annealing Tm (℃)
TAC4	CAAATGCTGTTGTCTGCTT	TATGGTTCAACTACATTCCG	270 bp	52℃
NK1Ra	GGAATGGATTCGCTCATCACTT	TAACGGTGTTGAATGCGGAC	280 bp	58℃
NK1Rb	CGTGTCGTGTGCTACATCAACT	TCATTTTCACCACCTTCCGT	220 bp	53℃
NK2R	AGATGATGATAGTGGTGGTGAC	GCAGTAGAGATGGGGTTGTA	177 bp	52℃
NK3Ra1	ATCGTAGTGGTGACCTTTGCC	CTCCATTCGTGAGAGCGTGTA	315 bp	60℃
NK3Ra2	CCGCCTTCAACACGCTCATCAA	GGAAAGCCAAAATCACCGCCAA	246 bp	52℃
NK3Rb	GCCAAGAGAAAGGTTGTGAAGA	GTGTACATGCTGCTCTGGCG	330 bp	56℃
β-actin	CTGGTATCGTGATGGACTCT	AGCTCATAGCTCTTCTCCAG	280 bp	56℃

Supplementary Table S2. Primer sequences and PCR conditions for real-time PCR for selected gene targets in grass carp

Gene	Forward primer	Reverse primer	Product size (bp)	Annealing Tm (℃)
UTS1	CTGACCACCCACATCCCCCT	AGTTCCGCCTGTTCCCTTTG	250 bp	59℃
CART2	CGCTGCTGCTCTGCTTGT	GCGGACAATCGCACATCT	280 bp	58℃
NMB1	AGCCTTGACTTGACTGAACTAA	CGGAACTTTAGGGCGGAT	220 bp	53℃
PRL	CTCAGCACCTCTCTCACCAATGA	GCGGAAGCAGGACAACAGAAAATG	400bp	60℃
SLα	ACCCACTGTACTTCAATCTCC	CGTCGTAACGATCAAGAGTAG	283 bp	58℃
β-actin	CTGGTATCGTGATGGACTCT	AGCTCATAGCTCTTCTCCAG	280 bp	56℃

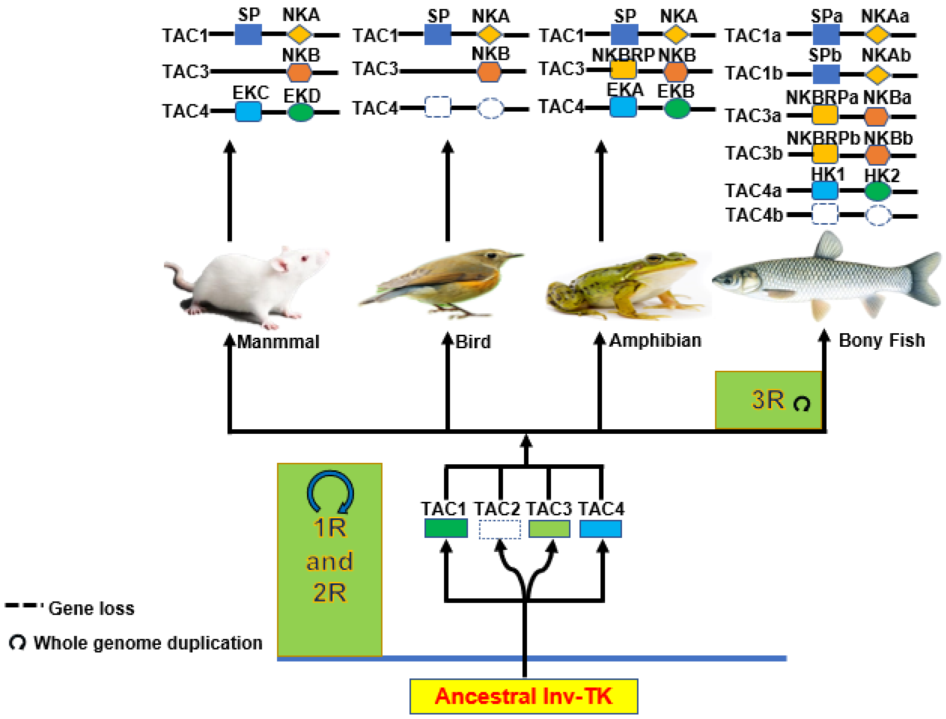

Supplemental Figure S1. The speculative evolutionary process of several rounds of genome replication of tachykinin family. Peptides and genes are displayed by colored labels. The path of evolution is marked by arrows.

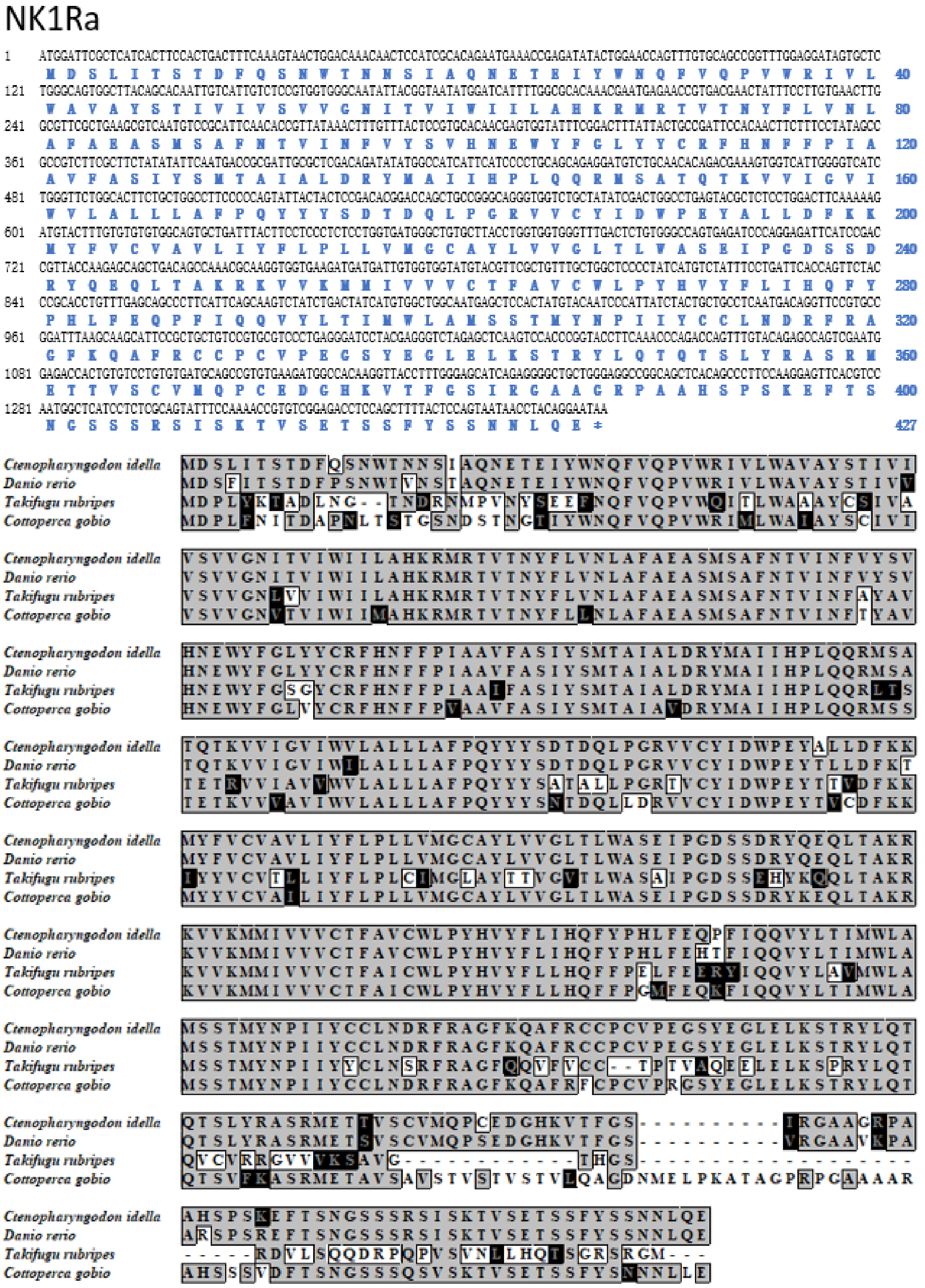

Supplemental Figure S2. **Molecular cloning and sequence alignment of grass carp NK1Ra. (A)** Nucleotide and deduced amino acid sequences of grass carp NK1Ra. Numbering of the deduced amino acid sequences begins with the first methionine of the ORF to the right of each line. Nucleotide numbers are to the left of each line. **(B)** Protein sequence alignment of grass carp NK1Ra with that of other vertebrates using Clustal-W algorithm with MacVector program. The conserved a.a. residues are boxed in grey.

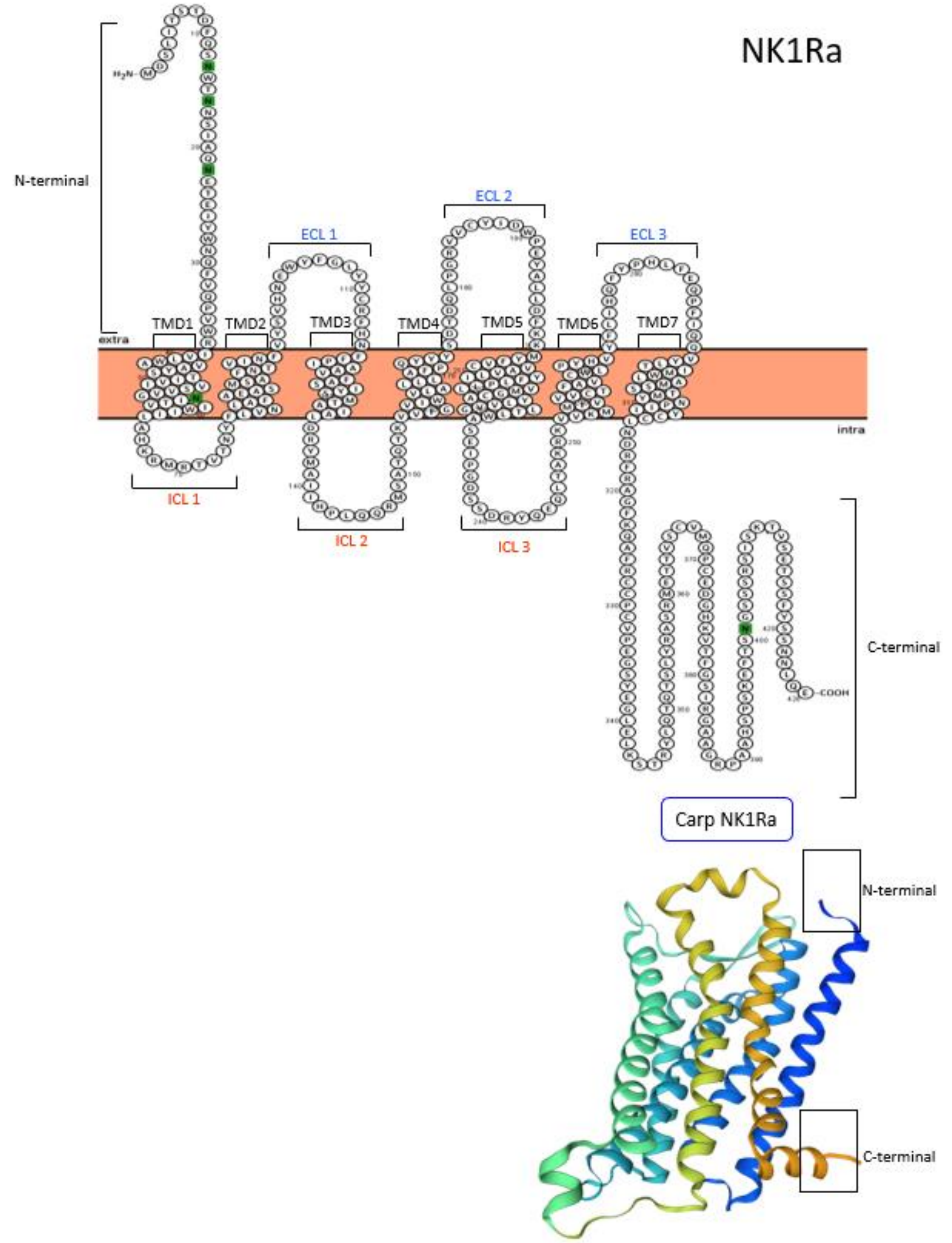

Supplemental Figure S3. **Sequence analysis of grass carp NK1Ra. (A)** Snake diagram of grass carp NK1Ra with Protter program. The seven transmembrane domains, three intracellular domains and three extracellular domains are labeled as TMD 1-7, ICL1-3 and ECL1-3, respectively. Sequence identities of transmembrane domains and extracellular domains, intracellular domains, N-termini and C-termini between grass carp and other vertebrates were showed in the Table. **(B)** 3-D protein model of grass carp NK1Ra using SWISS-MODEL program. The location of N-terminal and C-terminal were highlighted, respectively. The amino acids with hydrophobic side chains are colored blue, while those with hydrophilic side chains are colored red.

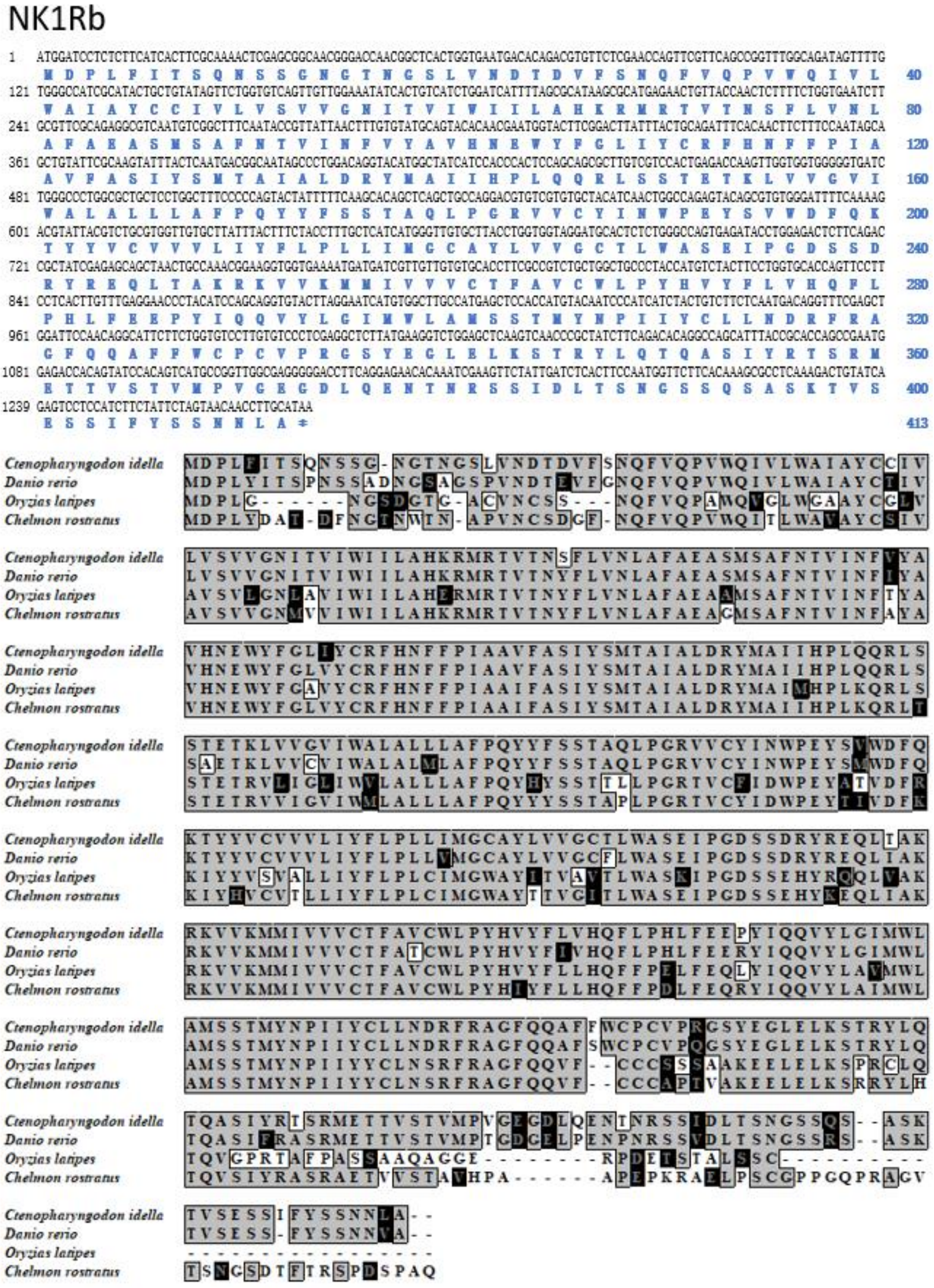

Supplemental Figure S4. **Molecular cloning and sequence alignment of grass carp NK1Rb. (A)** Nucleotide and deduced amino acid sequences of grass carp NK1Rb. Numbering of the deduced amino acid sequences begins with the first methionine of the ORF to the right of each line. Nucleotide numbers are to the left of each line. **(B)** Protein sequence alignment of grass carp NK1Ra with that of other vertebrates using Clustal-W algorithm with MacVector program. The conserved a.a. residues are boxed in grey.

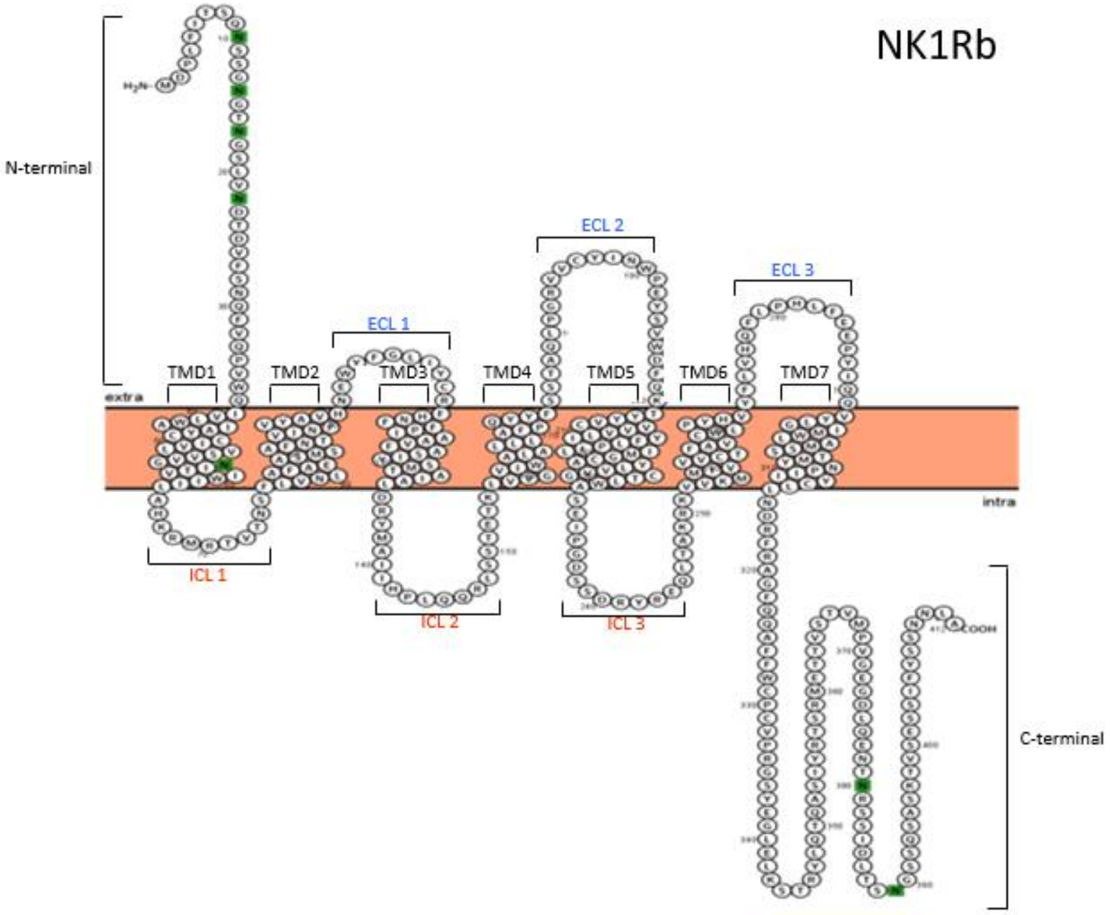

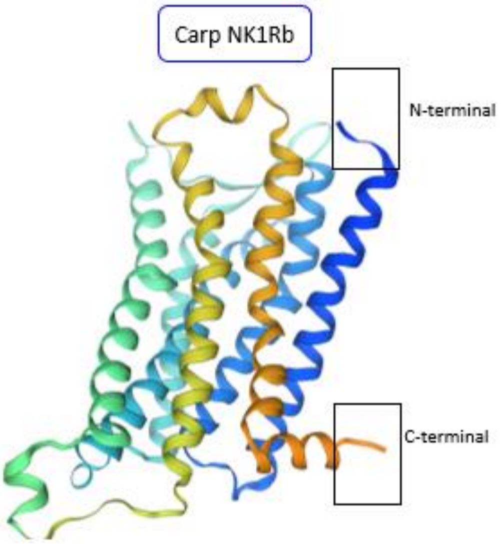

Supplemental Figure S5. **Sequence analysis of grass carp NK1Rb. (A)** Snake diagram of grass carp NK1Rb with Protter program. The seven transmembrane domains, three intracellular domains and three extracellular domains are labeled as TMD 1-7, ICL1-3 and ECL1-3, respectively. Sequence identities of transmembrane domains and extracellular domains, intracellular domains, N-termini and C-termini between grass carp and other vertebrates were showed in the Table. **(B)** 3-D protein model of grass carp NK1Rb using SWISS-MODEL program. The location of N-terminal and C-terminal were highlighted, respectively. The amino acids with hydrophobic side chains are colored blue, while those with hydrophilic side chains are colored red.

NK2R

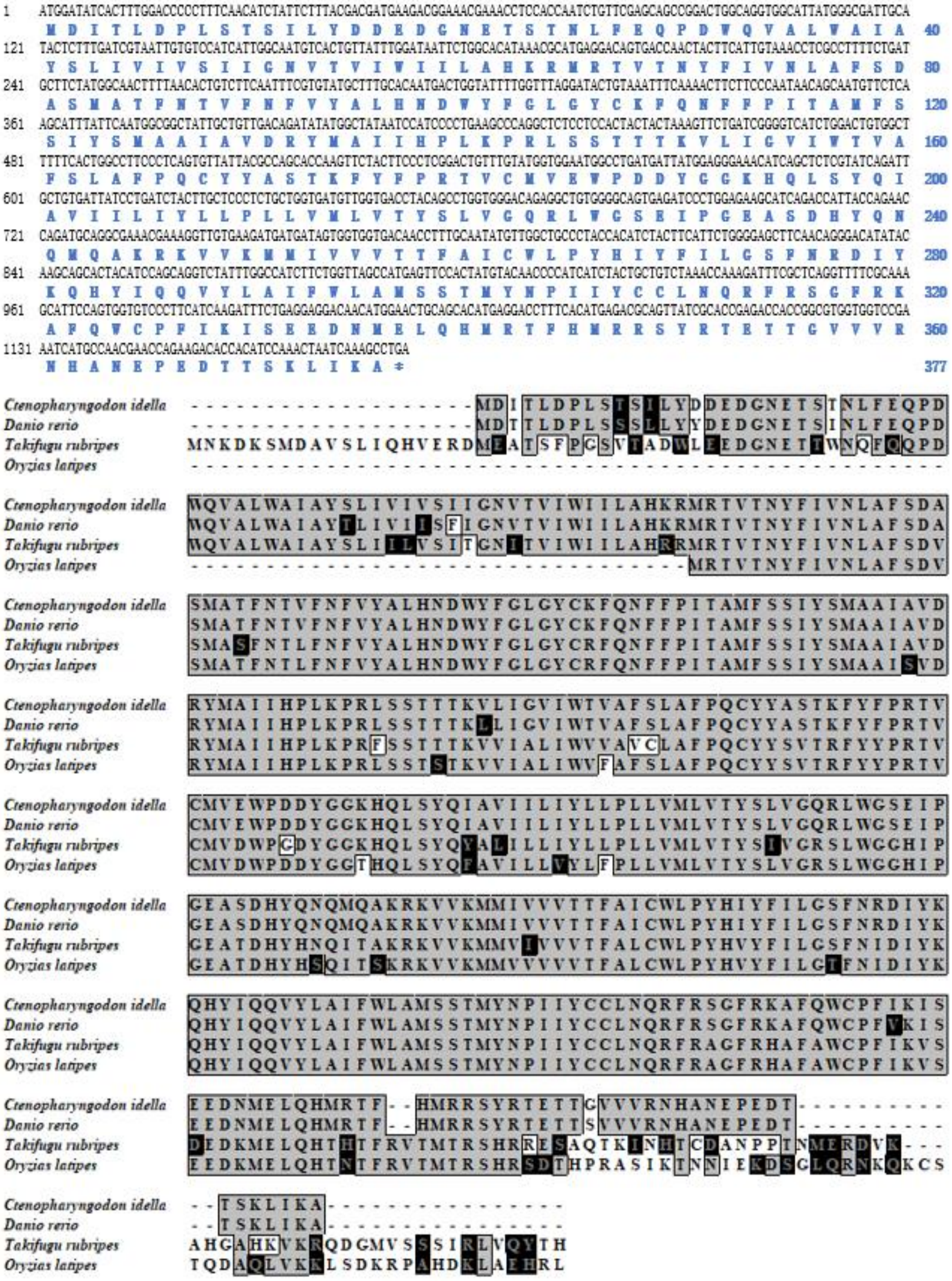

Supplemental Figure S6. **Molecular cloning and sequence alignment of grass carp NK2R. (A)** Nucleotide and deduced amino acid sequences of grass carp NK2R. Numbering of the deduced amino acid sequences begins with the first methionine of the ORF to the right of each line. Nucleotide numbers are to the left of each line. **(B)** Protein sequence alignment of grass carp NK1Ra with that of other vertebrates using Clustal-W algorithm with MacVector program. The conserved a.a. residues are boxed in grey.

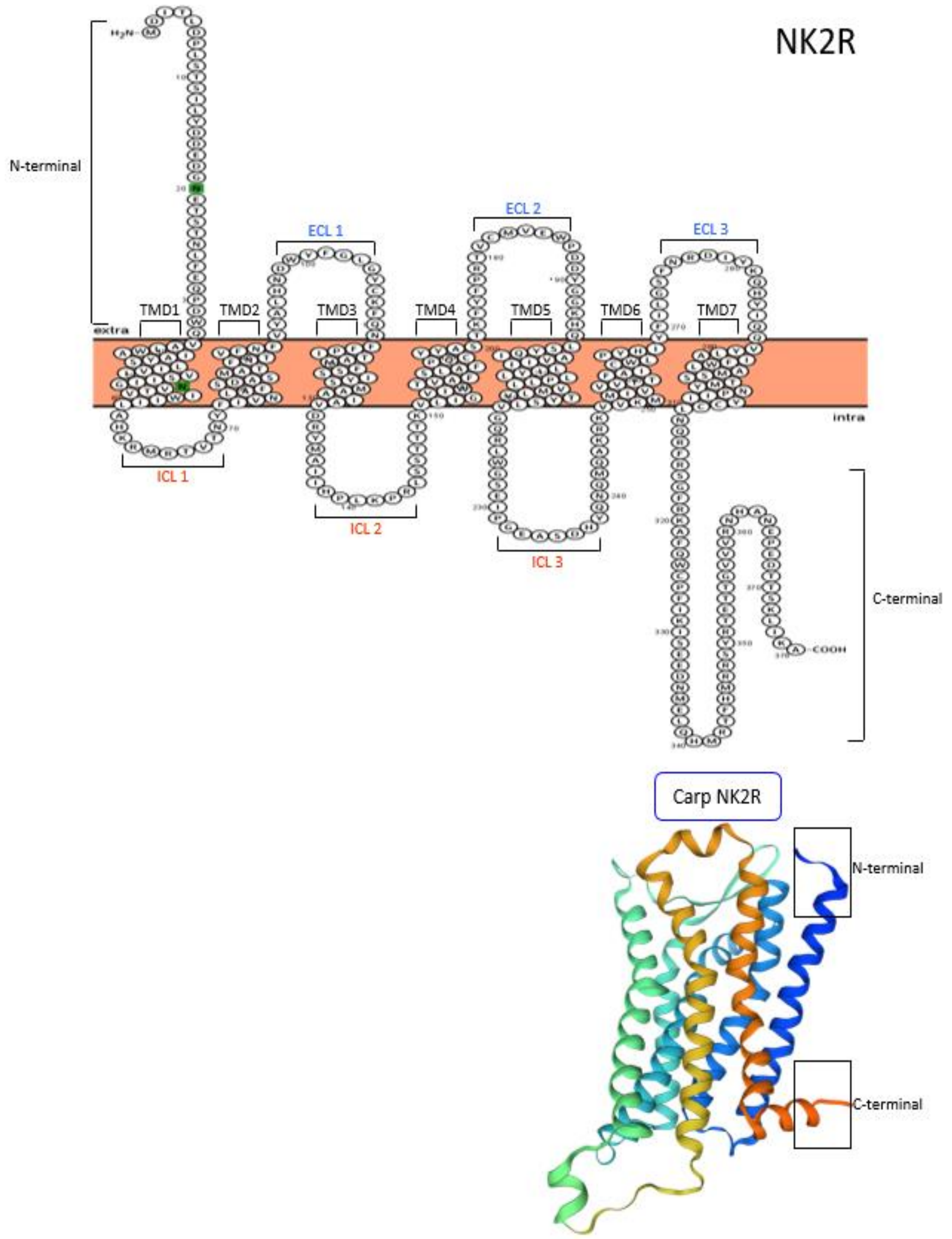

Supplemental Figure S7. Sequence analysis of grass carp NK2R. (A) Snake diagram of grass carp NK2R with Protter program. The seven transmembrane domains, three intracellular domains and three extracellular domains are labeled as TMD 1-7, ICL1-3 and ECL1-3, respectively. Sequence identities of transmembrane domains and extracellular domains, intracellular domains, N-termini and C-termini between grass carp and other vertebrates were showed in the Table. **(B)** 3-D protein model of grass carp NK2R using SWISS-MODEL program. The location of N-terminal and C-terminal were highlighted, respectively. The amino acids with hydrophobic side chains are colored blue, while those with hydrophilic side chains are colored red.

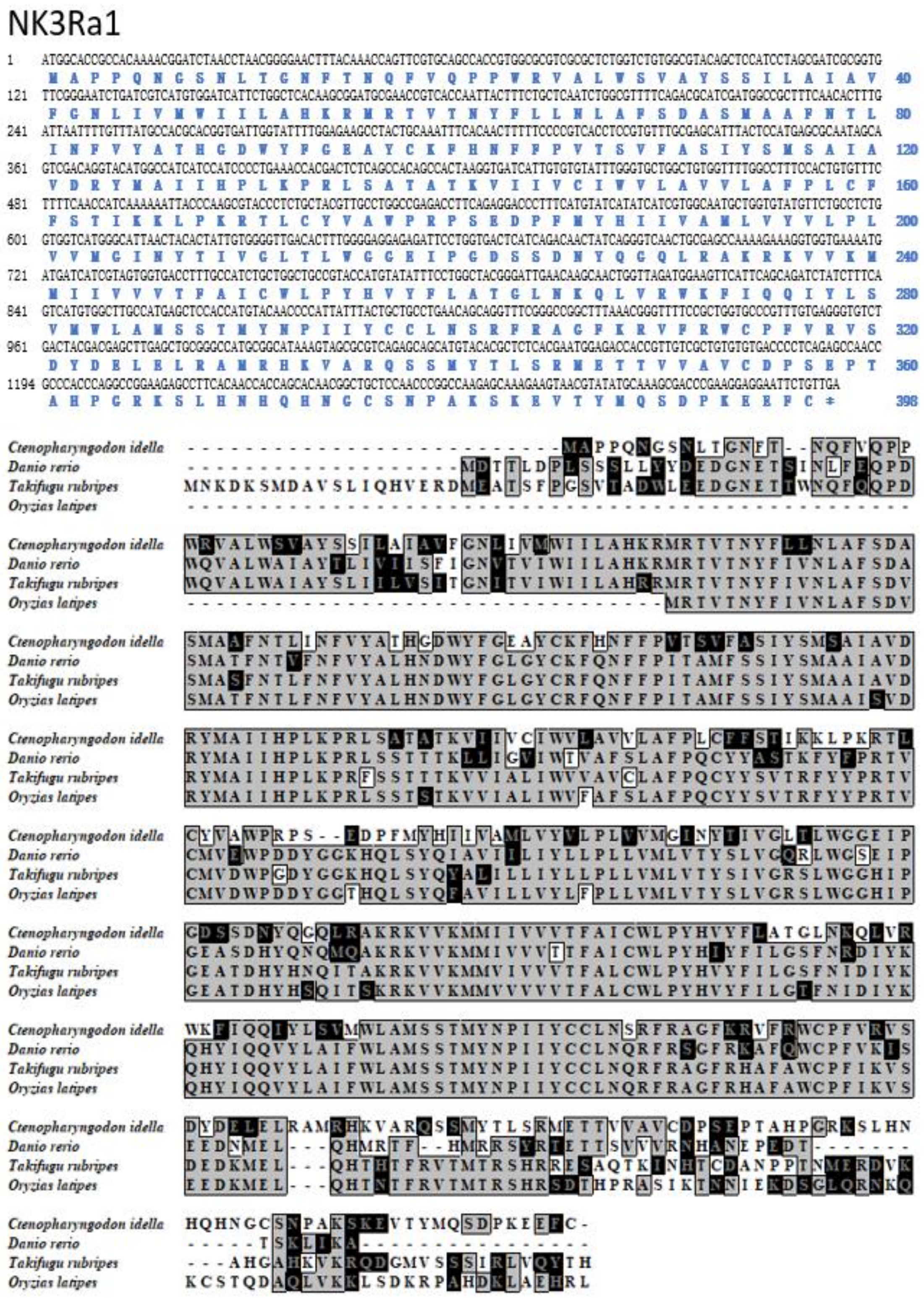

Supplemental Figure S8. **Molecular cloning and sequence alignment of grass carp NK3Ra1. (A)** Nucleotide and deduced amino acid sequences of grass carp NK3Ra1. Numbering of the deduced amino acid sequences begins with the first methionine of the ORF to the right of each line. Nucleotide numbers are to the left of each line. **(B)** Protein sequence alignment of grass carp NK1Ra with that of other vertebrates using Clustal-W algorithm with MacVector program. The conserved a.a. residues are boxed in grey.

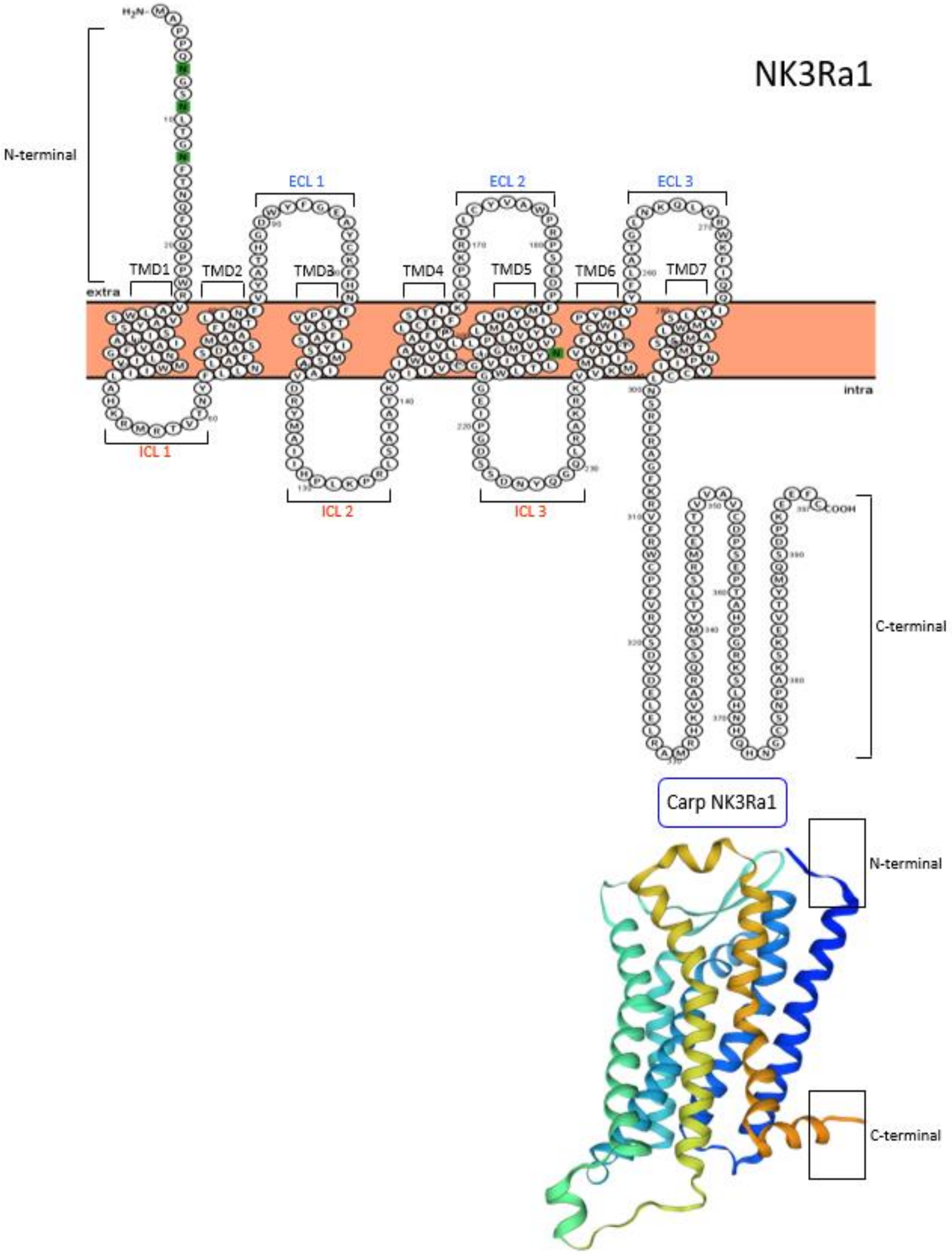

Supplemental Figure S9. **Sequence analysis of grass carp NK3Ra1. (A)** Snake diagram of grass carp NK3Ra1 with Protter program. The seven transmembrane domains, three intracellular domains and three extracellular domains are labeled as TMD 1-7, ICL1-3 and ECL1-3, respectively. Sequence identities of transmembrane domains and extracellular domains, intracellular domains, N-termini and C-termini between grass carp and other vertebrates were showed in the Table. **(B)** 3-D protein model of grass carp NK3Ra1 using SWISS-MODEL program. The location of N-terminal and C-terminal were highlighted, respectively. The amino acids with hydrophobic side chains are colored blue, while those with hydrophilic side chains are colored red.

NK3Ra2

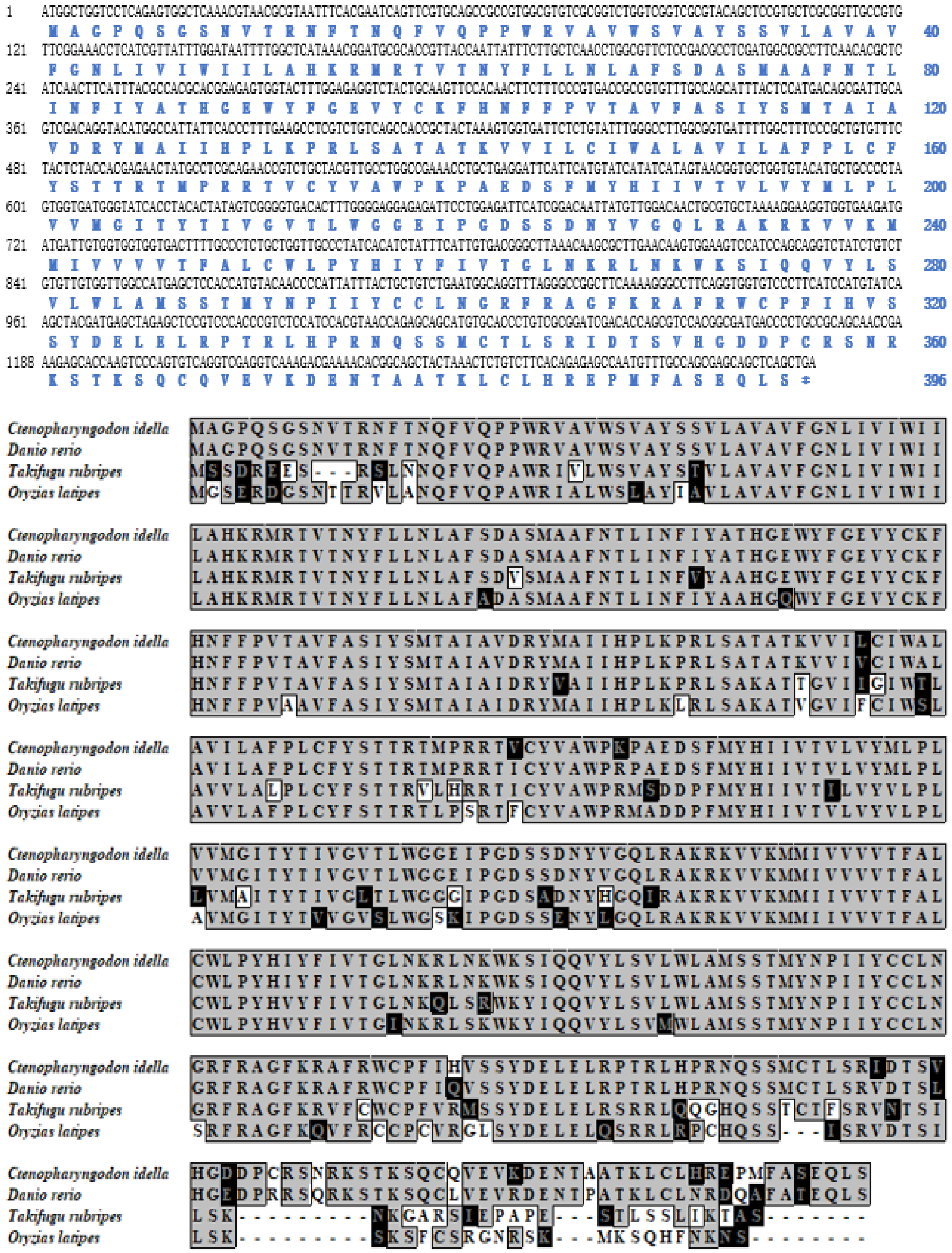

Supplemental Figure S10. **Molecular cloning and sequence alignment of grass carp NK3Ra2. (A)** Nucleotide and deduced amino acid sequences of grass carp NK3Ra2. Numbering of the deduced amino acid sequences begins with the first methionine of the ORF to the right of each line. Nucleotide numbers are to the left of each line. **(B)** Protein sequence alignment of grass carp NK1Ra with that of other vertebrates using Clustal-W algorithm with MacVector program. The conserved a.a. residues are boxed in grey.

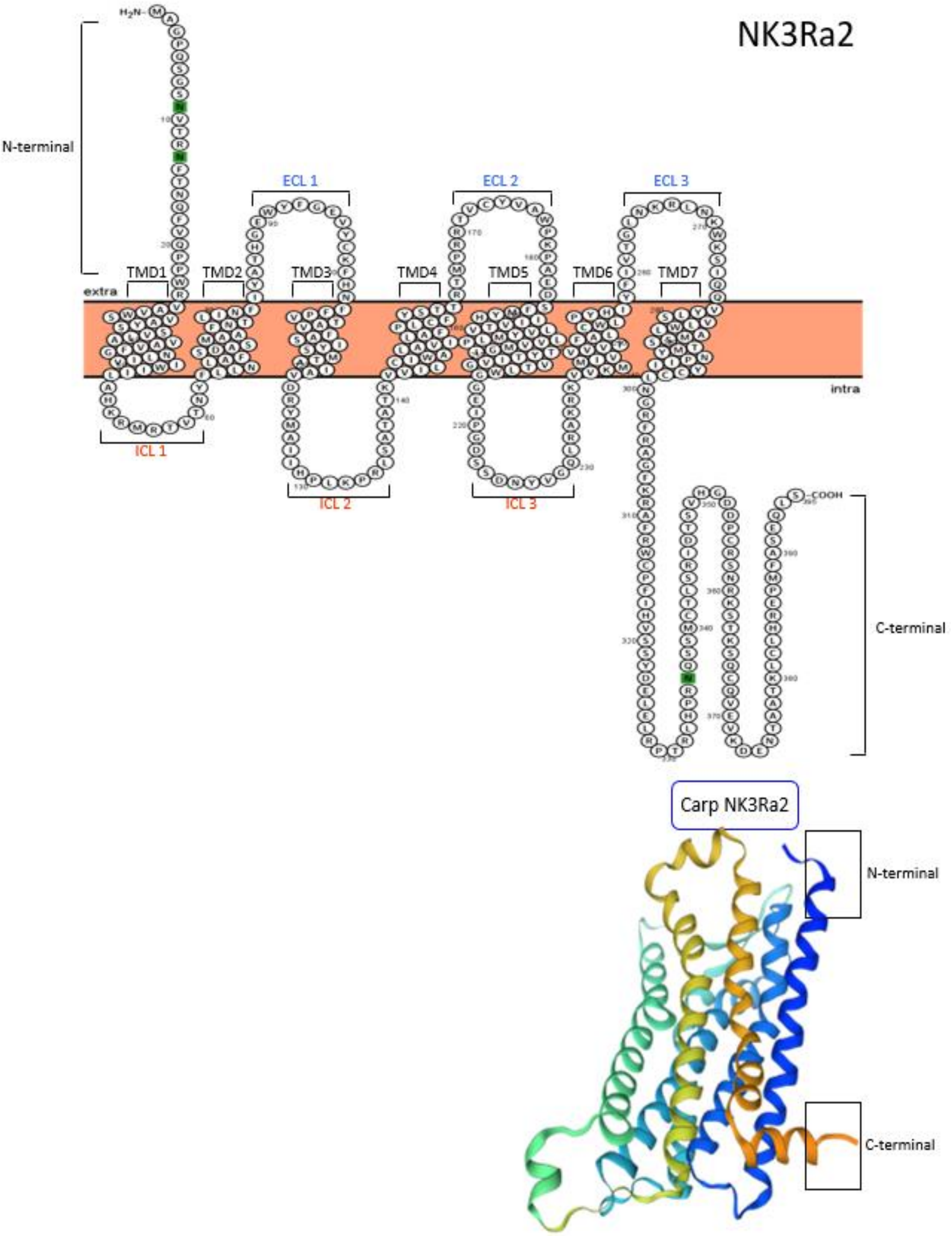

Supplemental Figure S11. **Sequence analysis of grass carp NK3Ra2. (A)** Snake diagram of grass carp NK3Ra2 with Protter program. The seven transmembrane domains, three intracellular domains and three extracellular domains are labeled as TMD 1-7, ICL1-3 and ECL1-3, respectively. Sequence identities of transmembrane domains and extracellular domains, intracellular domains, N-termini and C-termini between grass carp and other vertebrates were showed in the Table. **(B)** 3-D protein model of grass carp NK3Ra2 using SWISS-MODEL program. The location of N-terminal and C-terminal were highlighted, respectively. The amino acids with hydrophobic side chains are colored blue, while those with hydrophilic side chains are colored red.

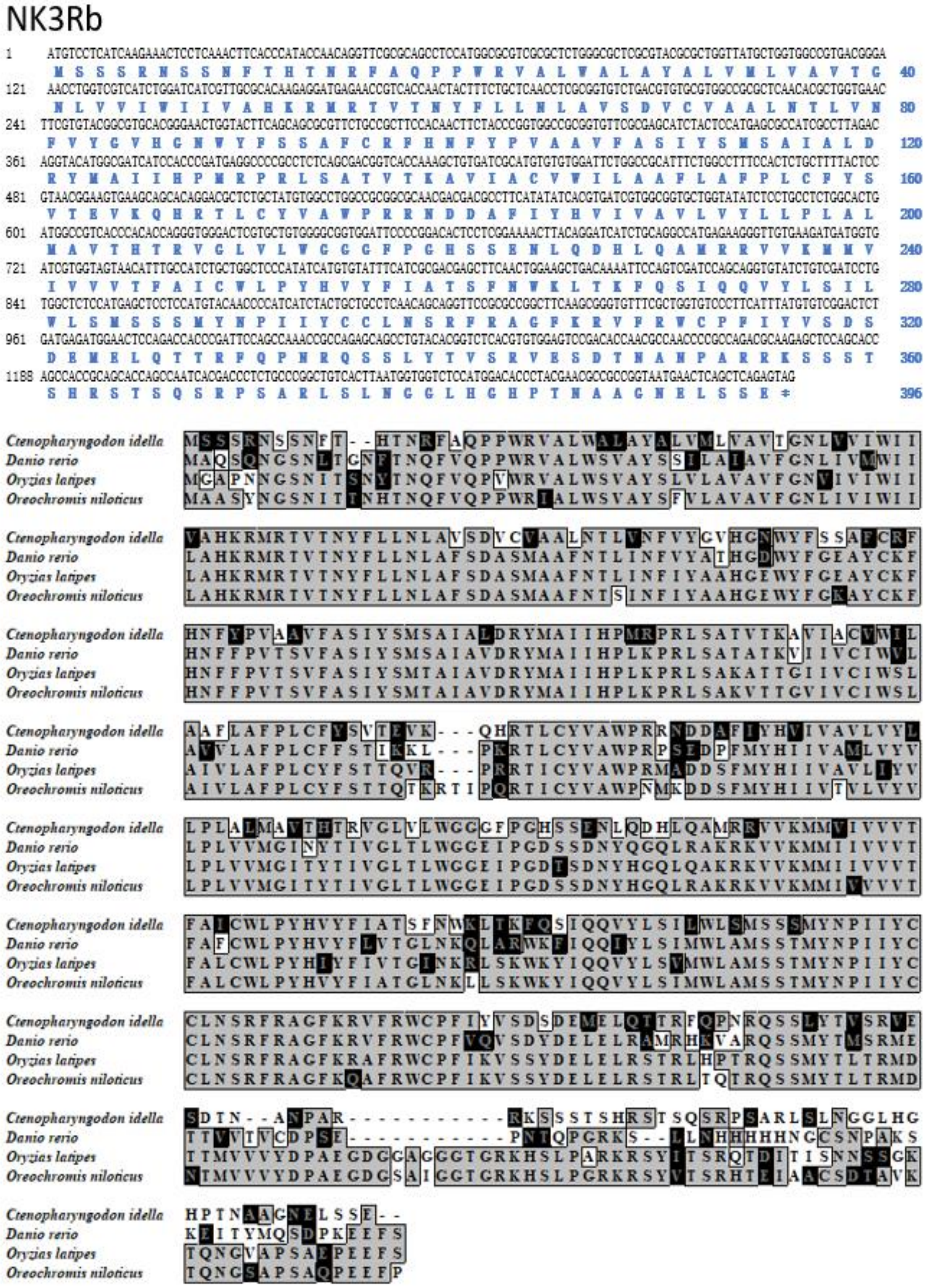

Supplemental Figure S12. **Molecular cloning and sequence alignment of grass carp NK3Rb. (A)** Nucleotide and deduced amino acid sequences of grass carp NK3Rb. Numbering of the deduced amino acid sequences begins with the first methionine of the ORF to the right of each line. Nucleotide numbers are to the left of each line. **(B)** Protein sequence alignment of grass carp NK1Ra with that of other vertebrates using Clustal-W algorithm with MacVector program. The conserved a.a. residues are boxed in grey.

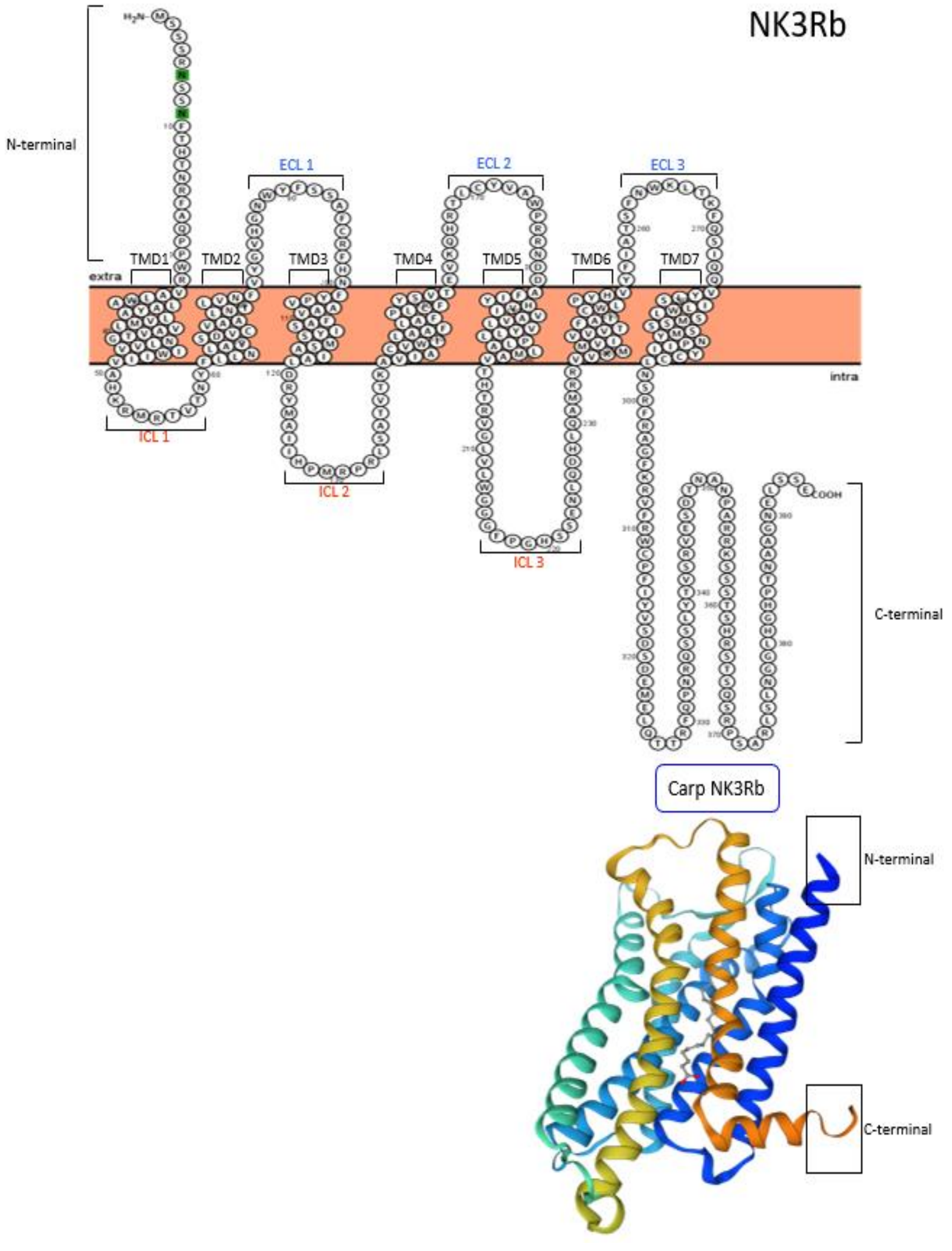

Supplemental Figure S13. **Sequence analysis of grass carp NK3Rb. (A)** Snake diagram of grass carp NK3Rb with Protter program. The seven transmembrane domains, three intracellular domains and three extracellular domains are labeled as TMD 1-7, ICL1-3 and ECL1-3, respectively. Sequence identities of transmembrane domains and extracellular domains, intracellular domains, N-termini and C-termini between grass carp and other vertebrates were showed in the Table. **(B)** 3-D protein model of grass carp NK3Rb using SWISS-MODEL program. The location of N-terminal and C-terminal were highlighted, respectively. The amino acids with hydrophobic side chains are colored blue, while those with hydrophilic side chains are colored red.

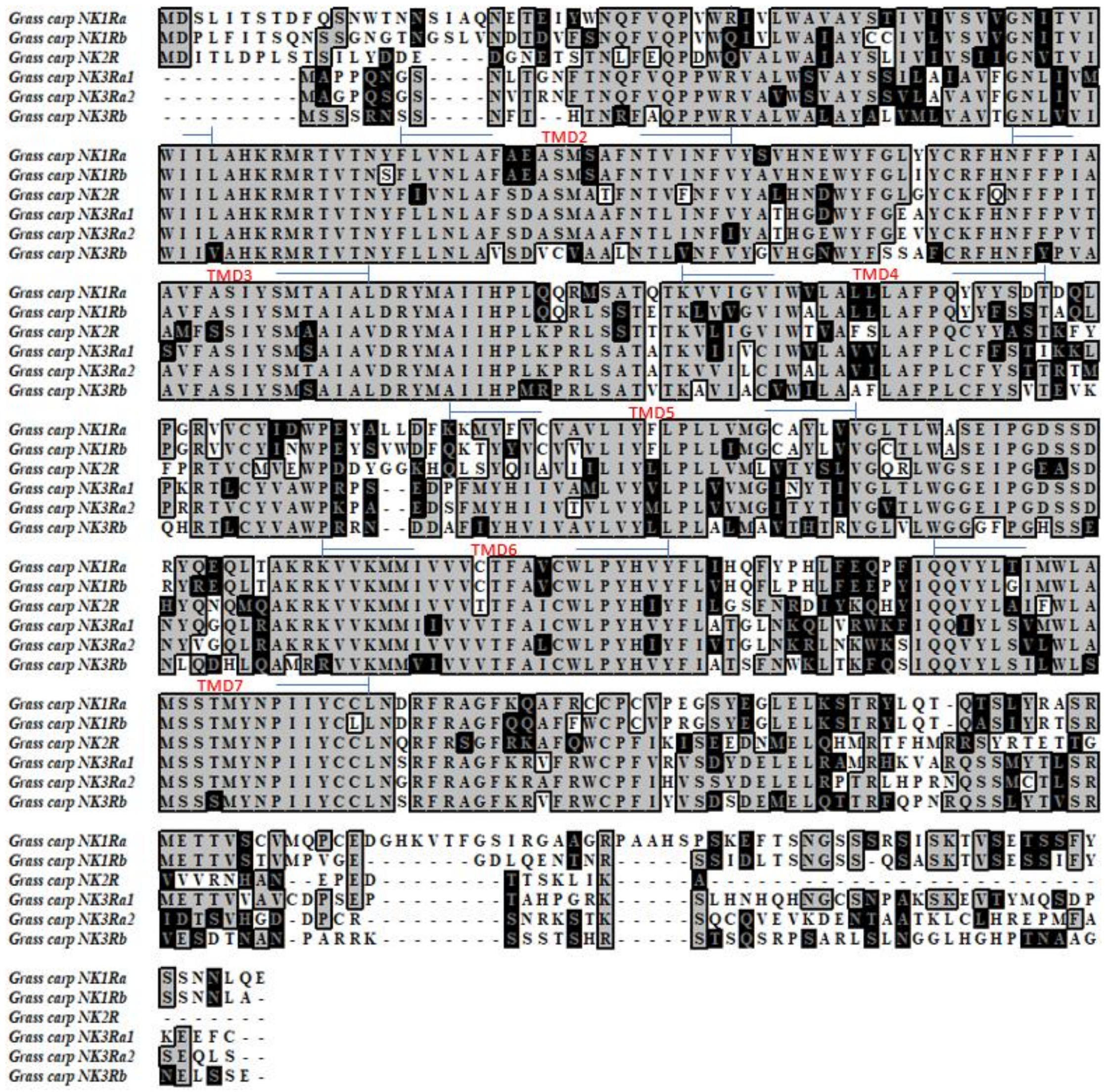

Supplemental Figure S14. **Sequence alignment of grass carp NK1Ra, NK1Rb, NK2R, NK3Ra1, NK3Ra2 and NK3Rb.** Protein sequence alignment of grass carp NK1Ra, NK1Rb, NK2R, NK3Ra1, NK3Ra2 and NK3Rb using Clustal-W algorithm with MacVector program. The lower conserved a.a. residues are boxed in grey where higher are in black. The seven transmembrane domainsare labeled as TMD1-7, respectively.
